# Flavonoids from *Chroogomphus rutilus* attenuate DSS-induced colitis, exhibiting anti-inflammatory, antioxidant, and gut microbiota-modulating effects

**DOI:** 10.3389/fmicb.2026.1886708

**Published:** 2026-08-10

**Authors:** Dan Wang, Feng Luo, Mingyi Zhou, Hongli Shang, Xizhu Wang

**Affiliations:** Liaoning Provincial Professional Technology Innovation Center of Meat Processing and Quality-Safety Control, College of Food and Health, Jinzhou Medical University, Jinzhou, Liaoning, China

**Keywords:** *Chroogomphus rutilus*, colitis, flavonoid, gut microbiota, network pharmacology

## Abstract

**Introduction:**

*Chroogomphus rutilus* (CR) is an edible mushroom valued for its bioactive polysaccharides and flavonoids. It possesses antioxidant and anti-inflammatory properties; however, whether it can confer protection against the onset of ulcerative colitis (UC) remains unclear.

**Methods:**

Initial network pharmacology analysis was performed to evaluate the potential influence of the flavonoid fraction of CR (CRF) on UC-related targets involved in inflammatory signaling and oxidative stress. The effects of CRF pretreatment were subsequently investigated in a mouse model of dextran sulfate sodium (DSS)-induced colitis. Mice received oral CRF pretreatment at doses of 100 or 400 mg/kg, followed by induction of acute colitis with 3% DSS.

**Results:**

CRF pretreatment suppressed colonic proinflammatory cytokine expression and preserved antioxidant defense. 16S rRNA gene sequencing revealed that CRF modulated the gut microbiota composition by suppressing potentially harmful bacteria, such as *Bacteroides* and *Erysipelatoclostridium*, and enriching beneficial genera including *Bifidobacterium*, *Dubosiella*, and *Faecalibaculum*.

**Discussion:**

These correlative findings suggest that CRF pretreatment attenuates DSS-induced colitis, and that this attenuation is accompanied by reduced inflammation, alleviated oxidative stress, and microbiota remodeling. The data support the hypothesis that CRF acts through multi-target preventive effects, highlighting its potential as a candidate functional food ingredient for further development.

## 1 Introduction

Inflammatory bowel disease (IBD) is a chronic, relapsing disorder of the gastrointestinal tract, primarily encompassing ulcerative colitis (UC) and Crohn’s disease (Kim et al., 2019). The global incidence of IBD has steadily increased over the past two decades (Ng et al., 2017). The etiology of UC remains unknown. The disease primarily affects the colon and rectum, with common symptoms including abdominal pain, bloody stools, persistent diarrhea, and weight loss (Su et al., 2021). The pathogenesis is not fully clear; however, systemic inflammation, oxidative stress, gut barrier damage, and gut dysbiosis seem to be closely linked to UC development (Li et al., 2023). Drugs such as mesalazine, glucocorticoids, and immunomodulators can relieve symptoms (Deng et al., 2025); however, their long-term use often causes intolerable side effects (Kobayashi et al., 2020). These limitations have driven interest in relatively safe bioactive compounds from natural sources.

Natural products from plants and fungi have attracted increasing attention in UC research (Jin et al., 2025; Wu et al., 2025). Among them, flavonoids are of particular interest because they target multiple pathological processes in colitis, including the suppression of inflammatory signaling, enhancement of antioxidant defense, preservation of intestinal barrier integrity, and modulation of the gut microbiota. For instance, flavonoids from *Potentilla anserina* L. alleviate oxidative stress through the Kelch-like ECH-associated protein 1–nuclear factor erythroid 2–related factor 2 signaling pathway, and those from Quzhou Aurantii Fructus suppress phosphoinositide 3-kinase (PI3K)/AKT-mediated inflammation and improve microbial homeostasis (Gao et al., 2025; Wang et al., 2025). Flavonoid extracts from *Malus spectabilis* flowers reduce colonic inflammation by inhibiting nuclear factor kappa beta (NF-κB)/mitogen-activated protein kinase (MAPK) signaling, increasing the expression of tight-junction proteins, and rebalancing gut microbial communities (Nong et al., 2024). In addition, flavonoids, such as rutin and apigenin, exert protective effects against UC by regulating the NOD-like receptor protein 3 (NLRP3) inflammasome and modulating the 5’ AMP-activated protein kinase/NF-κB/NLRP3 signaling network (Zhao et al., 2024; Zhou et al., 2025). Overall, these studies suggest that flavonoids deserve further investigation as multi-target agents for UC.

Edible macrofungi represent a promising source of flavonoids. The wild mushroom *Chroogomphus rutilus* (CR) exhibits anti-inflammatory, antioxidant, and hypoglycemic activities, which are primarily attributed to its polysaccharides and polyphenols (Guo et al., 2024; Zhang et al., 2020). However, the flavonoid constituents of CR (CR flavonoids, CRF) and their potential role in UC remain largely unexplored.

Based on this information, the present study aimed to investigate whether the flavonoid fraction of *Chroogomphus rutilus* (CRF) could prevent dextran sulfate sodium (DSS)-induced colitis and explore its effects on inflammation, oxidative stress, intestinal barrier function, and gut microbiota composition. We first applied network pharmacology to predict candidate targets and then evaluated the effects of CRF pretreatment in a DSS-induced colitis model of mice.

## 2 Materials and methods

### 2.1 Reagents and materials

Dextran sulfate sodium (DSS; molecular weight: 36,000–50,000 Da) was obtained from Yeasen Biotechnology Co., Ltd. (Shanghai, China). Mesalazine enteric-coated tablets were purchased from Sunflower Pharmaceutical Group (Heilongjiang, China). Specific assay kits for measuring colonic oxidative stress parameters, including total antioxidant capacity (T-AOC), superoxide dismutase (SOD), catalase (CAT), glutathione peroxidase (GSH-Px), malondialdehyde (MDA), and myeloperoxidase (MPO), were acquired from Nanjing Jiancheng Bioengineering Institute (Nanjing, China) and used according to the manufacturer’s instructions. Serum levels of tumor necrosis factor-*α* (TNF-*α*), interleukin-6 (IL-6), and interleukin-1*β* (IL-1*β*) were quantified using enzyme-linked immunosorbent assay (ELISA) kits purchased from Solarbio Science & Technology Co., Ltd. (Beijing, China). Rutin (purity: 98.65%) was obtained from Chengdu Maidesheng Technology Co., Ltd. (Chengdu, China).

### 2.2 Extraction and characterization of CRF

Based on the method of Chang et al. (2024) with minor modifications, vacuum-microwave-dried CR powder was mixed with 75% ethanol (v/v) at a sample-to-solvent ratio of 1:20 (g/mL). The mixture was subjected to ultrasonic extraction (28 kHz, 60 °C) for 30 min. The resulting extract was filtered and freeze-dried to obtain the crude flavonoid fraction (CRF). The CRF was preliminarily characterized by liquid chromatography–tandem mass spectrometry (LC–MS/MS) using an ACQUITY UPLC HSS T3 column (1.8 μm, 2.1 × 100 mm) maintained at 40 °C. The mobile phase consisted of 0.1% formic acid in water (A) and 0.1% formic acid in acetonitrile (B), delivered at a flow rate of 0.4 mL/min. The gradient elution program was as follows: 0–1.5 min, 5% B; 1.5–2.5 min, 5% → 10% B; 2.5–14 min, 10% → 40% B; 14–24 min, 40% → 95% B; 24–27 min, 95% B; 27–27.1 min, 95% → 5% B; 27.1–30 min, 5% B. Mass spectrometric detection was performed on an AB SCIEX TripleTOF 5,600 system equipped with an electrospray ionization (ESI) source, operating in both positive and negative ion modes. Information-dependent acquisition (IDA) was used with the following settings: survey scan range m/z 50–1,200; collision energy, 30 eV; maximum 15 MS/MS spectra per cycle; intensity threshold, 100 cps. The ion source parameters were set as follows: nebulizer gas, 60 psi; auxiliary gas, 60 psi; curtain gas, 35 psi; source temperature, 550 °C; ion spray voltage, 5,500 V (positive mode) and −4,500 V (negative mode). The corresponding LC–MS/MS data are provided in Supplementary information. The total flavonoid purity of CRF was approximately 65.2%.

### 2.3 Determination of total flavonoid content

Total flavonoid content was determined using a NaNO₂–Al(NO₃)₃–NaOH colorimetric method with rutin as the standard. Briefly, an aliquot of CRF solution was sequentially mixed with 5% NaNO₂, 10% Al(NO₃)₃ and 4% NaOH. After standing for 15 min, the absorbance was measured at 510 nm (Liu et al., 2025). Results were expressed as milligrams of rutin equivalents per gram of extract (mg RE/g).

### 2.4 Network pharmacology study

Chemical structures of CRF constituents in SMILES format were retrieved from PubChem (https://pubchem.ncbi.nlm.nih.gov/), and candidate protein targets were predicted computationally using the SwissTargetPrediction server (http://www.swisstargetprediction.ch/). Target information of all components was merged and saved. UC targets were retrieved from GeneCards (https://www.genecards.org/), OMIM (https://omim.org/), and CTD (https://ctdbase.org/) databases using the keyword “colitis.” The overlapping targets were imported into the STRING database to construct a protein–protein interaction (PPI) network, using a minimum required interaction score of 0.4 and excluding disconnected nodes. Cytoscape was employed to visualize the PPI network and identify topological hub proteins based on centrality metrics (Liao et al., 2025). Functional enrichment of the key targets was explored through Gene Ontology (GO), Kyoto Encyclopedia of Genes and Genomes (KEGG) pathway analyses using an R package (https://www.bioconductor.org/). Enriched terms were presented using bar plots and bubble charts.

### 2.5 Animal experiments

Fifty male Kunming mice (6–8 weeks old; body weight: 20 ± 2 g) were purchased from the SPF-certified Experimental Animal Center of Jinzhou Medical University. Mice were acclimated for 7 days in a controlled SPF environment (22–26 °C; 44–45% relative humidity; 12:12 h light–dark cycle), with unrestricted access to autoclaved water and standard chow. Animals were individually housed in ventilated cages lined with autoclaved corn cob bedding.

**Figure 1:**
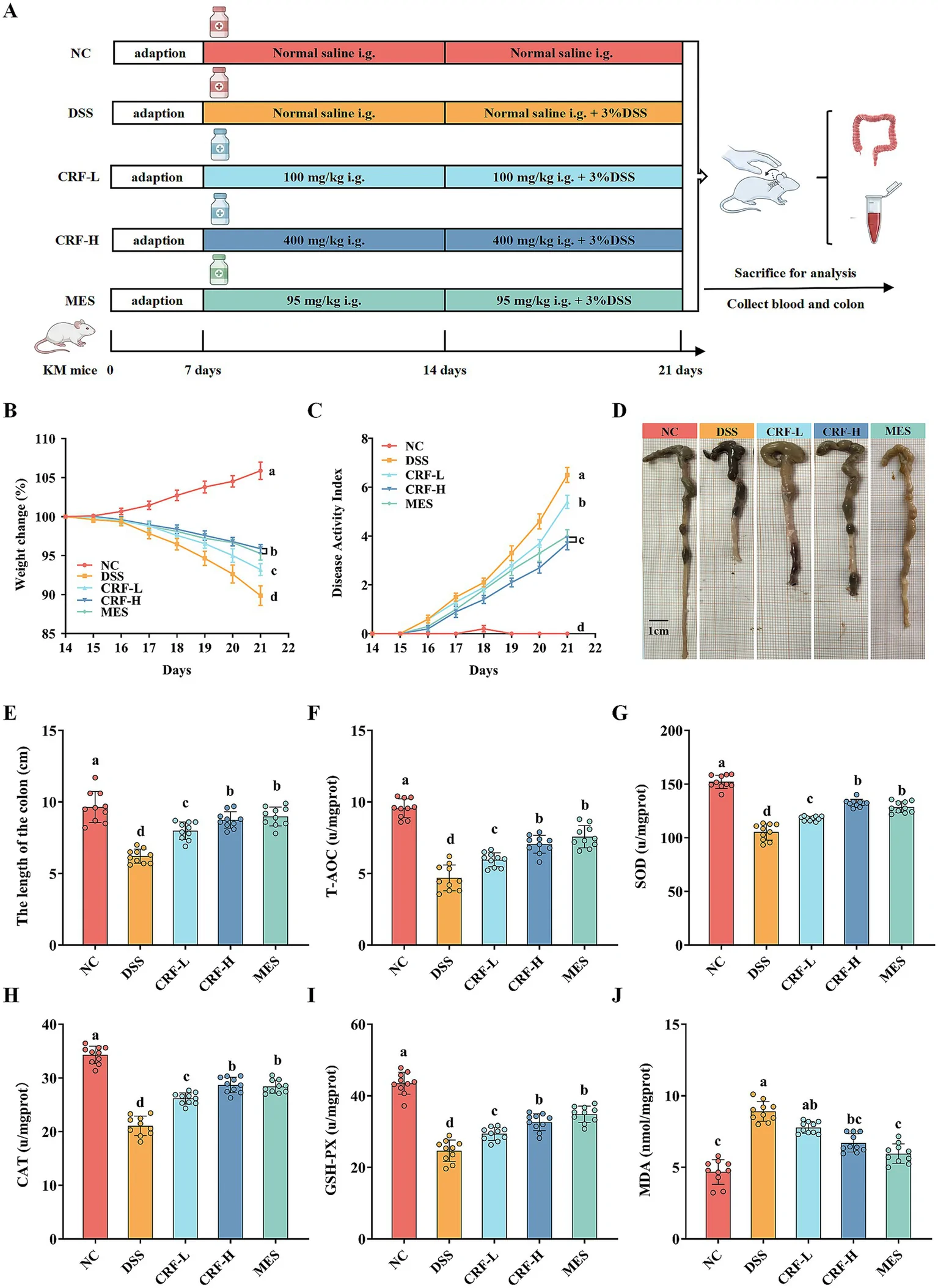
CRF pretreatment attenuates the onset of macroscopic manifestations and colonic oxidative stress in DSS-induced colitis. **(A)** Experimental design schematic. **(B)** BW changes. **(C)** DAI scores. **(D)** Representative images of colons from each group. **(E)** Colon length. Effects of CRF on the **(F)** T-AOC, **(G)** SOD activity, **(H)** CAT activity, **(I)** GSH-Px activity, and **(J)** MDA levels in mouse colonic tissue. For **(B–E)**, *n* = 10 mice per group. For **(F–J)**, *n* = 10 colonic tissue samples per group. All values represent the mean ± *SD*. Different lowercase letters denote statistically significant differences at *p* < 0.05.

Following acclimation, mice were randomly assigned to five groups (n = 10 per group) using a computer-generated random number table stratified by body weight: normal control (NC), DSS-induced colitis group (DSS), low-dose CRF (CRF-L; 100 mg/kg), high-dose CRF (CRF-H; 400 mg/kg), and mesalazine (MES; 95 mg/kg). The experimental design (Figure 1A) was adapted from a previous study, in which this MES dose was also used as a positive control (Fu et al., 2022). During the second and third weeks, the CRF-L and CRF-H groups received CRF (100 or 400 mg/kg) by oral gavage once daily, while the MES group received MES (95 mg/kg) via the same route. The control and DSS groups received an equal volume of saline vehicle. In the third week, colitis was induced in all groups except the NC group by providing 3% (w/v) DSS in drinking water for 7 consecutive days. At the end of the study, the mice were anesthetized, and blood samples were collected via retro-orbital puncture. Serum was separated and stored for subsequent cytokine analysis. The mice were then euthanized by cervical dislocation. All procedures were approved by the Institutional Animal Care and Use Committee of Jinzhou Medical University (approval No. 240146).

### 2.6 Disease activity index (DAI) assessment

The disease activity index (DAI) was calculated as the sum of scores for body weight loss, stool consistency, and fecal bleeding, based on previously described criteria (Mao et al., 2023; Zang et al., 2024). Body weight, stool consistency, and the presence of fecal blood were recorded daily during the DSS treatment period (Liu et al., 2025).

### 2.7 Determination of colonic biochemical parameters

Colon tissues were homogenized in ice-cold phosphate-buffered saline (PBS) at a ratio of 1:9 (w/v). The homogenates were centrifuged at 4 °C, and the resulting supernatants were collected. The levels of T-AOC, SOD, CAT, GSH-Px, MDA, and MPO were measured using commercial assay kits according to the manufacturer’s instructions.

### 2.8 Histological and immunohistochemical analyses

Colon segments (approximately 2 cm) were fixed in 4% paraformaldehyde for 24 h, then rinsed under running tap water. After fixation, the tissues were dehydrated through a graded ethanol series, cleared in xylene, embedded in paraffin, and sectioned. H&E and AB-PAS staining were performed following the manufacturer’s instructions. H&E-stained sections were examined under a light microscope as described in our previous work (Wang et al., 2021), and histological scoring was carried out according to Zhou et al. (2023). The distribution of goblet cells was assessed on AB-PAS-stained sections under a light microscope. Immunohistochemical staining was performed as reported by Ning et al. (2023).

### 2.9 16S rRNA sequencing of gut microbiota

Total DNA was extracted from fecal samples using the QIAamp PowerFecal Pro DNA Kit (Qiagen, Hilden, Germany) according to the manufacturer’s protocol, which includes a bead-beating step for mechanical lysis and inhibitor removal. Then, we amplified the V3–V4 region of the 16S rRNA gene by PCR using primers 338F and 806R. The PCR products were purified and quantified. Dual-index adapters were added to build sequencing libraries. Libraries that passed QC were subsequently sequenced on the Illumina NovaSeq 6,000 platform using a paired-end strategy. Raw reads were processed within the QIIME2 environment, which included quality-based filtering, denoising with DADA2, and chimera removal. The resulting amplicon sequence variants were assigned taxonomy using the Silva 138 database, which allowed us to carry out downstream diversity and differential analyses.

### 2.10 Statistical analysis

Data are presented as mean ± standard deviation. Examination of tissue sections and immunohistochemical images was performed using CaseViewer software. For the quantification of immunohistochemical staining, the area of positive staining was determined using ImageJ software. Data visualization was achieved using GraphPad Prism (v10.1.2). All statistical tests were performed with IBM SPSS Statistics (Version 27.0.0). For the visualized data, normality was first tested. If the data were normally distributed, homogeneity of variances was then examined. When equal variances were assumed, one-way ANOVA was performed; if a statistically significant difference was found, the Least Significant Difference post-hoc test was used for multiple comparisons. When equal variances could not be assumed, Tamhane’s T2 post-hoc test was applied. For data that did not meet the normality assumption, the Kruskal–Wallis non-parametric test was used directly. A *p*-value < 0.05 was considered statistically significant.

## 3 Results and discussion

### 3.1 Network pharmacology predicts the multi-target mechanism of CRF

**Figure 2:**
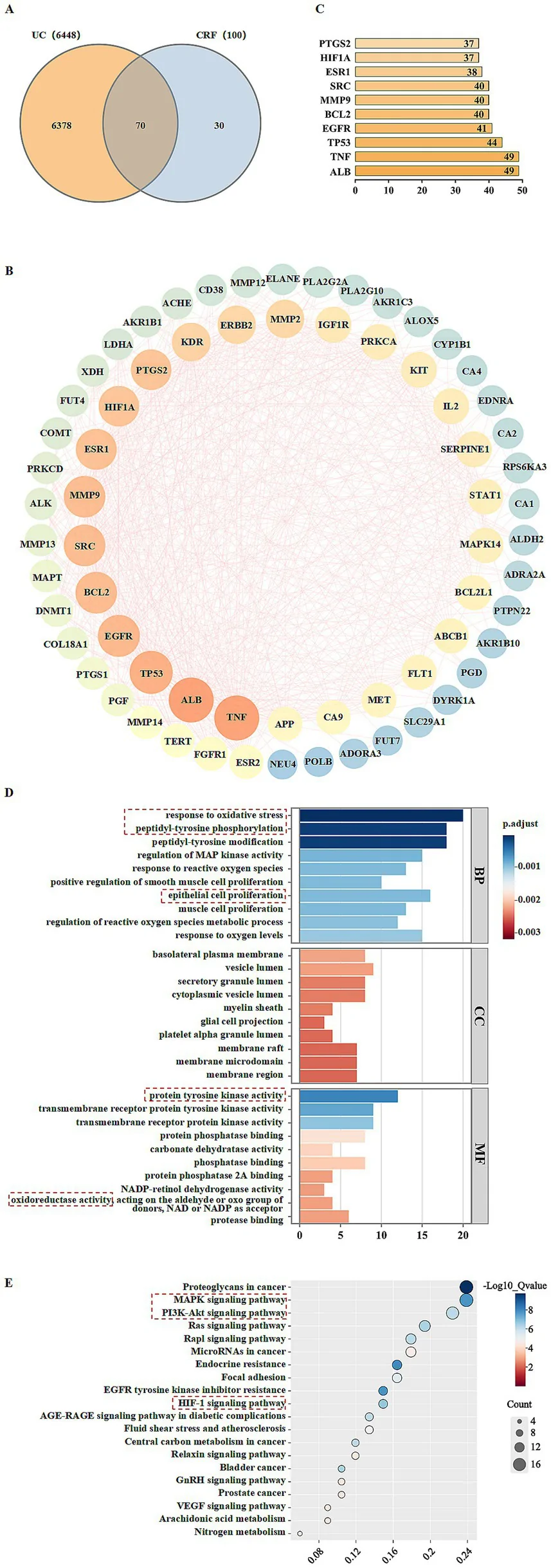
Network pharmacology analysis predicts the multi-target mechanisms and core signaling pathways of CRF against ulcerative colitis. **(A)** Venn diagram. **(B)** Bar chart of the top 10 core genes from the PPI network. **(C)** PPI network. **(D)** Top 10 enriched terms in GO analysis for BP, CC, and MF. **(E)** Top 20 signaling pathways identified by KEGG analysis.

The chemical composition of CRF was analyzed by mass spectrometry. Nineteen flavonoids and flavonols were identified in CRF. The total flavonoid content of CRF, determined spectrophotometrically with rutin as the standard, was 84.54 mg RE/g. Detailed chemical information and mass spectra are shown in Supplementary Table S1 and Supplementary Figure S1. To investigate whether CRF plays a preventive role in UC, we first used a network pharmacological approach. A Venn diagram identified 70 overlapping genes between the predicted targets of CRF and UC-related targets (Figure 2A). These targets may represent potential preventive candidates for CRF in the context of UC (Supplementary Table S2). Subsequently, these intersecting genes were input into the STRING database, and the PPI network was constructed and visualized using Cytoscape v.3.7.2. (Figure 2B). The resulting network comprised 68 nodes and 586 edges. The nodes represented the target proteins, whereas the edges reflected interactions among them. In the visualization, brighter colors and large node sizes indicated high degree values, suggesting high importance of the corresponding nodes within the network. As shown in Figure 2C, the key core targets were determined according to their degree values, with the five top being ALB, TNF, TP53, EGFR, and BCL2. Albumin, a key extracellular antioxidant, serves as a clinical marker to distinguish between active and quiescent ulcerative colitis (Tratenšek et al., 2024), and it reflects systemic oxidative stress via its free thiol status (Geertsema et al., 2024), pointing to an alleviation of pathological protein leakage. TNF acts as a key driver of gut inflammation. TP53 and BCL2 intricately regulate cellular apoptosis and genomic stability under chronic oxidative stress in the inflamed colon (Hirsch et al., 2021). EGFR is a pivotal mediator of epithelial proliferation and mucosal healing (Otte et al., 2023). To further elucidate the biological functions and potential molecular mechanisms, GO functional annotation and KEGG pathway enrichment analyses were performed. Subsequently, the 10 top genes were selected for further examination. Biological process (BP) analysis demonstrated that CRF was closely linked to the key pathological events of UC, particularly response to oxidative stress, peptidyl-tyrosine modification, and epithelial cell proliferation (Figure 2D). These results are consistent with the findings of previous PPI-network analysis. Regarding the cellular component, these intersection targets were predominantly localized in regions critical for cellular signal transduction. Furthermore, the molecular function outcomes revealed that these targets were involved in protein tyrosine kinase and oxidoreductase activities. According to the KEGG pathway analysis, the underlying mechanism was associated with core inflammatory and repair networks, primarily the MAPK, PI3K–AKT, and hypoxia-inducible factor 1 signaling pathways (Figure 2E). Based on these *in silico* predictions, we hypothesize that CRF exerts preventive effects by mitigating oxidative stress-induced inflammatory responses and promoting intestinal epithelial-cell proliferation and survival, thereby potentially accelerating mucosal healing. However, these predicted pathways have not yet been directly validated at the molecular level, and their involvement remains to be experimentally confirmed.

### 3.2 Establishment of a mouse model of experimental colitis

BW and DAI were recorded to evaluate the protective effect of CRF against DSS-induced colitis in mice. BW loss, diarrhea, fecal bleeding, and colon shortening are well-established indicators of colitis severity. DSS intervention induced significant BW loss (*p* < 0.05), elevated DAI scores (*p* < 0.05), and markedly shortened colon length (*p* < 0.05) compared to those observed in the NC group (Figures 1B–E). Consistent with previous findings, mice in the DSS group exhibited significantly shortened colons and a marked reduction in BW (Lin et al., 2024), indicating successful model establishment. Pretreatment with CRF and MES attenuated DSS-induced weight loss and colon shortening, and attenuated the DSS-induced increase in DAI scores, compared to those in the DSS group. Notably, the preventive effect of CRF-L was less pronounced than that of CRF-H, indicating that CRF exerted a dose-dependent suppressive effect on the development of DSS-induced colitis. These results are in line with the reported protective effects of chrysin, a flavonoid identified in our CRF extract (Supplementary Table S1). In a DSS-induced colitis model, chrysin treatment significantly reduced DAI scores, prevented BW loss, and ameliorated colon shortening (Yao et al., 2025). Therefore, chrysin may be a bioactive constituent contributing to the preventive benefits of CRF. Collectively, these results demonstrate that high-dose CRF pretreatment attenuates DSS-induced colitis, supporting its protective role against intestinal inflammation.

### 3.3 CRF mitigates oxidative stress in DSS-induced mice

Oxidative stress is a recognized risk factor for chronic inflammatory diseases and a driver of DNA-damaging inflammation, thereby contributing to UC progression. The antioxidant defense system that counteracts this stress relies on primary enzymatic antioxidants, including SOD, CAT, and GSH-Px. In this study, the activities of T-AOC, SOD, CAT, and GSH-Px in the DSS group were significantly lower than those in the NC group, consistent with previous studies (Qiao et al., 2025). Pretreatment with MES or CRF partially attenuated the DSS-induced decrease in these enzyme activities; nevertheless, the activities after pretreatment remained significantly lower than those in the NC group (Figure 1F–I, *p* < 0.05).

MDA is a product of lipid peroxidation and a reliable indicator of membrane oxidative damage (Ren et al., 2020). The MDA level in the DSS group was 8.9 ± 0.66 nmol/mg protein, which was the highest among all groups (Figure 1J). This indicates pronounced lipid peroxidation, consistent with previous reports (Huang et al., 2026). Pretreatment with MES and CRF significantly attenuated the DSS-induced elevation of MDA (*p* < 0.05). Similarly, a polyphenol-rich extract from green pea hull, in which catechin and its derivatives are among the major constituents, has been reported to restore colonic SOD, CAT, and T-AOC activities in mice with DSS-induced colitis (Su et al., 2021). Given the presence of catechin in our CRF extract (Supplementary Table S1), this flavonoid may partially account for preserving antioxidant enzyme activities observed in our study. Network pharmacology analysis predicted that this protective effect could involve the AGE–RAGE signaling pathway that is linked to oxidative stress. Taken together, these results indicate that CRF pretreatment attenuates oxidative stress-induced injury in DSS-induced colitis.

### 3.4 CRF prevents colonic histological damage in DSS-induced mice

**Figure 3:**
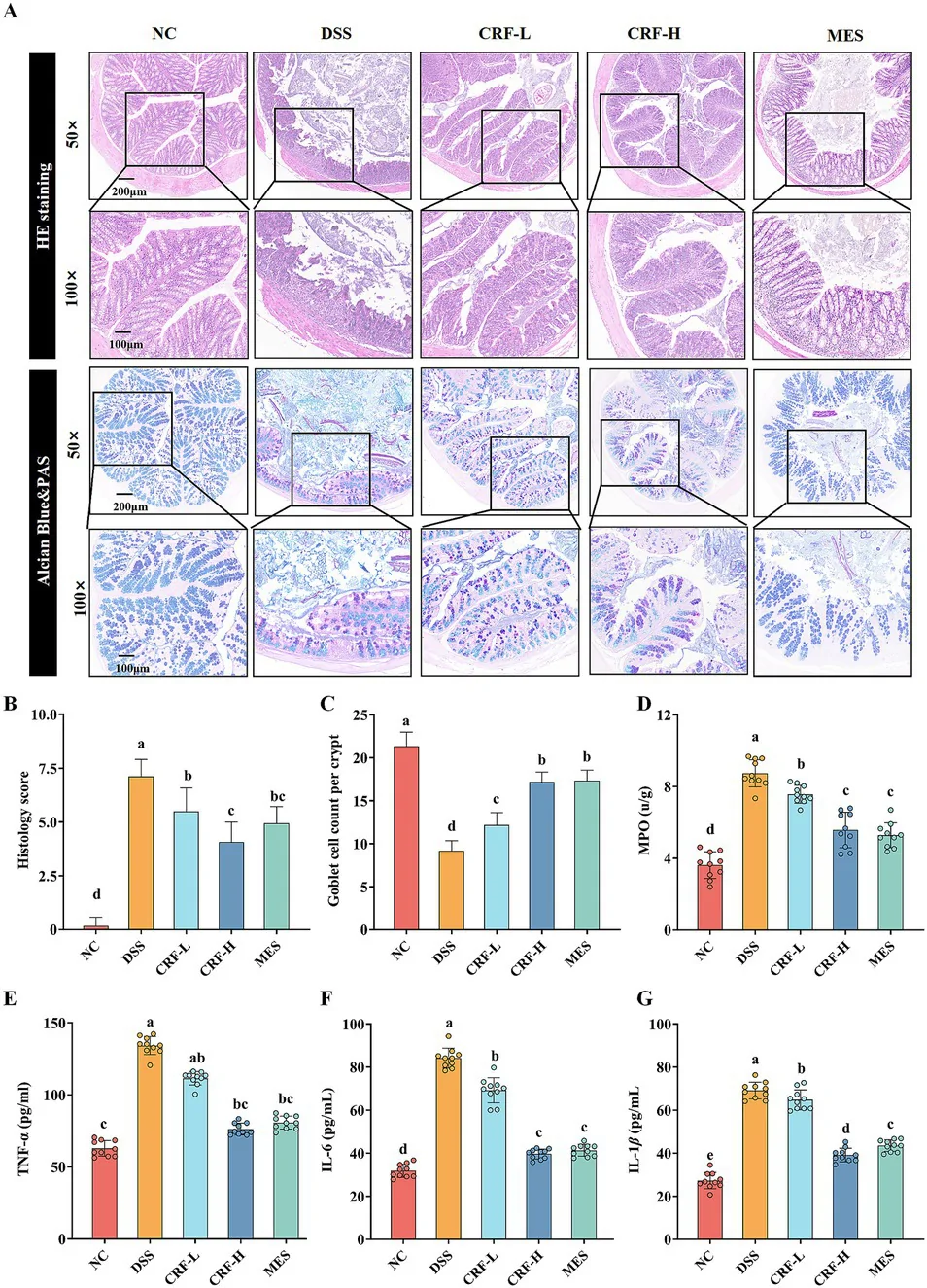
CRF pretreatment attenuates the onset of colonic histological damage and preserves mucosal integrity. **(A)** Histological features revealed by H&E and AB-PAS staining, scale bars: 200 μm (100×) and 100 μm (50×). **(B)** Semiquantitative scores of histopathological severity scored from H&E-stained sections (*n* = 6 mice/group). **(C)** Goblet cell count per crypt (*n* = 6 mice/group). **(D)** MPO enzyme activity in the colon (*n* = 10 mice/group). **(E)** Serum levels of TNF-*α*, **(F)** IL-6, and **(G)** IL-1*β* (*n* = 6 mice/group). Data are presented as mean ± SD. Groups labeled with different letters differ significantly (*p* < 0.05).

Oxidative stress impairs the intestinal barrier (Kleme and Levy, 2015), activates inflammatory pathways, and drives cellular apoptosis. These processes directly contribute to colonic mucosal injury and inflammatory responses in mice. We examined colonic tissue histology using H&E and AB-PAS staining (Figure 3A). In the NC group, the colonic architecture was intact with well-formed crypts, ordered cell layers, abundant goblet cells, and a clear mucosa–submucosa boundary. In contrast, the DSS group showed opposite trends with distorted crypts, severely damaged mucosal epithelium, decreased number of goblet cells, and a clear infiltration of inflammatory cells. These pathological features align with those previously reported (Deng et al., 2025), confirming successful model establishment. Pretreatment with MES or CRF attenuated DSS-induced colonic tissue damage, with CRF showing a dose-dependent effect. Histological scores and goblet cell count per crypt further supported these findings (Figures 3B,C).

MPO, a marker of neutrophil infiltration, was measured as an additional indicator of inflammation (Xu et al., 2021). DSS treatment significantly increased colonic MPO activity to 8.74 ± 0.76 U/g, compared with 3.62 ± 0.74 U/g in the NC group (*p* < 0.01; Figure 3D). This represents approximately a 141% increase. Pretreatment with MES or CRF significantly attenuated this increase, restoring MPO levels toward those of the NC group. Therefore, CRF pretreatment attenuates both oxidative/inflammatory responses and structural damage in the colon, thereby helping to preserve the mucosal barrier. Baicalin, a flavonoid identified in our CRF extract (Supplementary Table S1), ameliorates DSS-induced colitis by reducing pathological scores, maintaining goblet cell abundance, and restoring the expression of tight junction proteins claudin-1, ZO-1, and occludin (Liu et al., 2026). This protective effect has been attributed to the regulation of macrophage polarization via the JAK1/STAT1/SOCS1 pathway. These findings align with our observation that CRF pretreatment partially attenuates colonic tissue injury and support the hypothesis that baicalin may contribute to the barrier-protecting effects of CRF.

### 3.5 CRF suppresses systemic proinflammatory cytokine expression in DSS-induced mice

DSS-induced colitis elevates circulating proinflammatory cytokine levels, reflecting a systemic inflammatory response (Xing et al., 2024). Therefore, we measured serum levels of IL-1*β*, IL-6, and TNF-*α* as indicators of systemic inflammatory burden, whereas local colonic inflammation was assessed by measuring MPO activity (Section 3.4). DSS challenge led to markedly elevated serum levels of these cytokines (Figures 3E–G). Pretreatment with CRF, particularly at the high dose, significantly attenuated these elevations. This finding, together with the decreased colonic MPO activity and attenuation of histopathological damage, suggests that CRF pretreatment is accompanied by suppression of both local neutrophil infiltration and systemic inflammatory response. These observations are consistent with the network pharmacological prediction that CRF may target the TNF signaling pathway. Given that TNF-*α* is a key driver of inflammation (Chen J. et al., 2025), the observed reduction in serum TNF-*α* level might contribute to the downregulation of IL-1*β* and IL-6, thereby potentially interrupting the self-amplifying inflammatory cycle. We acknowledge that serum cytokine levels may not fully represent the local colonic milieu, as they can be influenced by systemic dilution and extra-intestinal sources. Therefore, changes in systemic cytokine levels reported here should be interpreted as indicators of overall inflammatory status; their direct mechanistic link to colonic pathology requires further investigation. Collectively, these observations identify the TNF signaling cascade as a potential pathway involved in the activity of CRF, and functional studies are needed to confirm its role in attenuating inflammatory responses.

### 3.6 CRF attenuates DSS-induced disruption of intestinal barrier integrity

**Figure 4:**
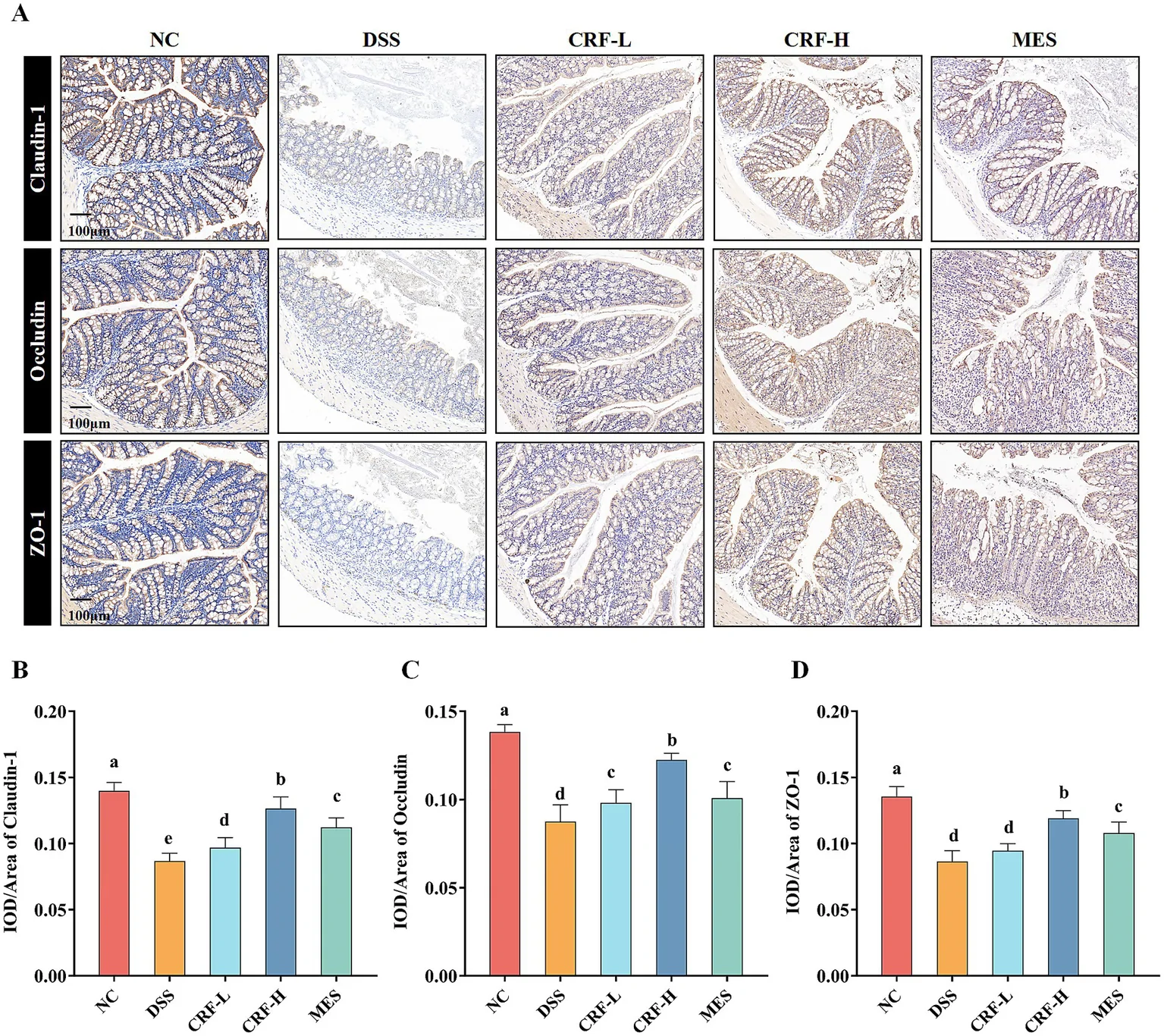
CRF pretreatment maintains intestinal barrier integrity by preserving the expression of colonic tight junction proteins. **(A)** Representative immunohistochemical images of tight junction proteins in colonic tissues, scale bars: 100 μm. **(B–D)** Expression levels of claudin-1, occludin, and ZO-1 in the colonic tissue (*n* = 6 mice/group). Values are shown as mean ± SD. Distinct lowercase letters mark significant differences between groups (*p* < 0.05).

The colonic mucosal barrier is interconnected with the intestinal barrier to form a coordinated protective system. Tight junction proteins, including the transmembrane proteins claudin-1 and occludin and the cytoplasmic scaffold protein ZO-1, serve as key indicators of intestinal barrier function. To evaluate the preventive potential of CRF in restoring the impaired intestinal barrier in murine colitis, we examined colonic expression of the tight junction proteins claudin-1, occludin, and ZO-1 by immunohistochemistry (Figure 4). Relative to that observed in the NC group, mice in the DSS group exhibited a significant reduction in the immunohistochemical staining intensity of these proteins (*p* < 0.05), as previously observed (Guo et al., 2025). Pretreatment with MES or CRF significantly attenuated DSS-induced decrease in the expression of these proteins. However, the levels of these proteins were markedly low compared to that observed in the NC group, (*p* < 0.05) indicating that the preventive effect on intestinal barrier function was partial. Phloretin, a dihydrochalcone flavonoid identified in our CRF extract (Supplementary Table S1), protects intestinal barrier integrity in DSS-induced colitis by upregulating claudin-1 and ZO-1 expression, an effect linked to its regulatory action on the gut microbiota (Wu et al., 2019). This observation is consistent with the partial restoration of tight junction proteins expression by CRF in our study and suggests that phloretin may at the bioactive constituent contributing to the barrier-protective effects of CRF. This finding aligns with the network pharmacological prediction that the core targets of CRF are enriched in pathways closely related to cell adhesion such as Focal adhesion and the Rap1 signaling pathway. Therefore, CRF pretreatment partially prevents DSS-induced disruption of intestinal barrier integrity.

### 3.7 CRF regulates gut microbiota dysbiosis

**Figure 5:**
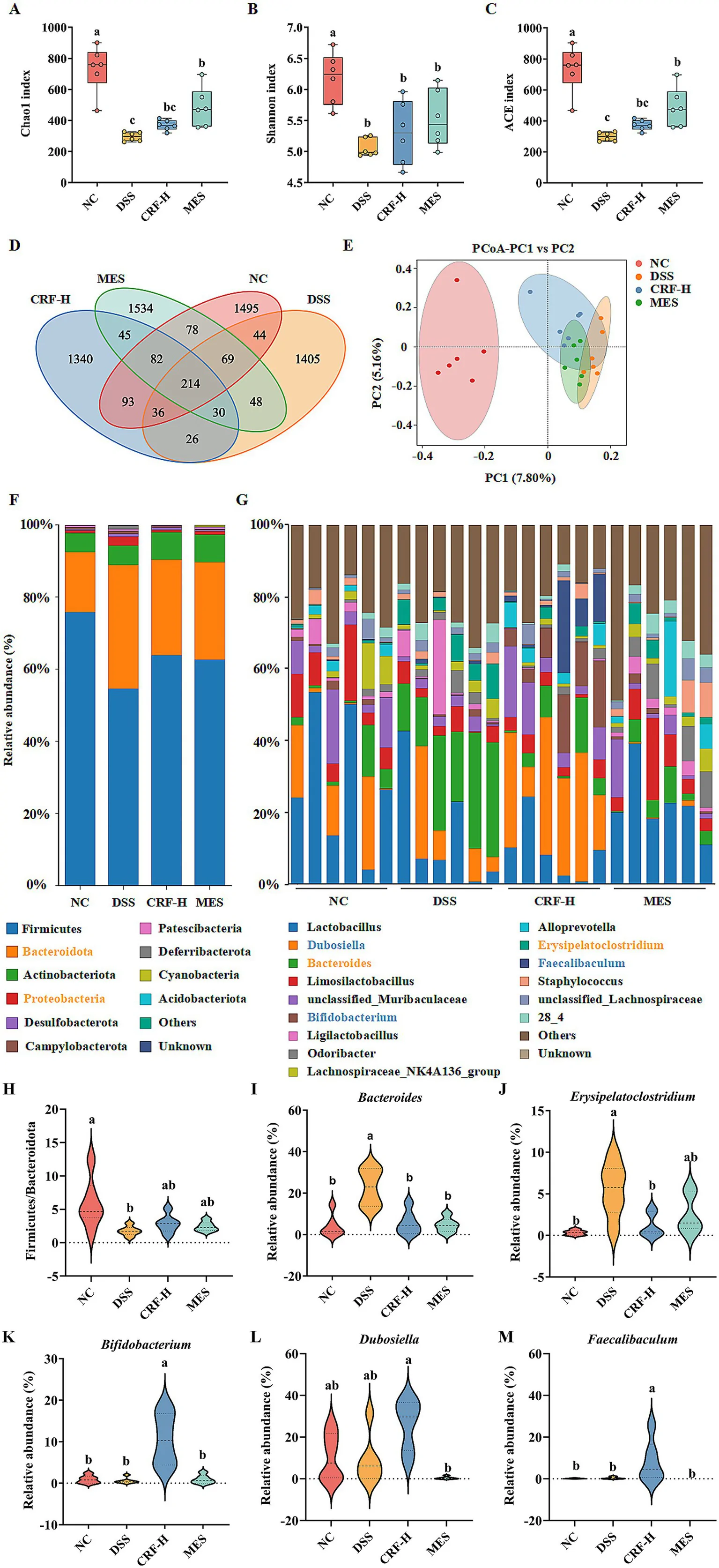
CRF-H prophylaxis rescues DSS-induced gut microbiota dysbiosis and restores global microbial community structure. Alpha-diversity indices: **(A–C)** Chao1, **(B)** Shannon, and **(C)** ACE. **(D)** Number of operational taxonomic units per group. **(E)** Principal coordinate analysis. **(F)** Relative microbial abundance at the phylum level. **(G)** Relative microbial abundance at the genus level. **(H)** Ratio of *Firmicutes* to *Bacteroidota* (F/B) at the phylum level. Relative abundances of the following at the genus level: **(I–M)** *Bacteroides*, **(J)** *Erysipelatoclostridium*, **(K)** *Bifidobacterium*, **(L)** *Dubosiella*, and **(M)** *Faecalibaculum*. Data are presented as mean ± SD (*n* = 6). Groups labeled with different letters differ significantly (*p* < 0.05).

Alpha diversity indices, including Chao1, ACE, Shannon, Simpson, Pielou, inverse Simpson, and Good’s coverage, reflect community richness and evenness. In this study, the Chao1, Shannon, and ACE indices were used to evaluate alpha diversity. Compared with the NC group, the Chao1, Shannon, and ACE indices were significantly lower in the DSS group (Figures 5A–C). Consistent with the results reported by Luo et al. (2025), the DSS group exhibited significantly reduced Chao1 and Shannon indices, indicating diminished microbial richness and alpha diversity. However, CRF-H pretreatment attenuated this disturbance and partially prevented the loss of microbial richness. The NC group harbored 1,495 unique OTUs, whereas the DSS group contained 1,405 unique OTUs (Figure 5D). The MES and CRF-H groups contained 1,534 and 1,340 unique OTUs, respectively. We performed Principal Coordinates Analysis (PCoA) to assess *β-*diversity across the groups. PCoA revealed a clear separation between the NC and DSS groups along PC1, which accounted for 7.80% of the total variance (Figure 5E). This separation indicates that DSS induced a substantial disturbance in the gut microbiota. A similar separation between DSS-treated and healthy mice was reported by Zhang et al. (2025). The confidence ellipses of the DSS, MES, and CRF-H groups overlapped substantially, indicating that the structural shift induced by DSS remained the dominant variation. The CRF-H group was positioned further to the left along PC1 than the DSS and MES groups, closer to the NC baseline level. Overall, CRF-H pretreatment did not completely prevent DSS-induced microbial dysbiosis but partially attenuated the disruption.

We also assessed the gut microbiota at the phylum and genus levels. At the phylum level, the microbial community was dominated by *Firmicutes*, *Bacteroidota*, *Actinobacteriota*, and *Proteobacteria*, which collectively represented over 90% of the total sequences (Figure 5F). Among these phyla, *Firmicutes* and *Bacteroidota* together accounted for over 80% of the sequenced reads, reflecting their high prevalence and compositional dominance within the gut ecosystem. Compared with the NC group, the DSS group displayed increased relative abundances of *Bacteroidota* and *Proteobacteria*, along with a decreased relative abundance of *Firmicutes*, resulting in a reduction in the *Firmicutes*/*Bacteroidota* (F/B) ratio. Although the F/B ratio is not a definitive biomarker for ulcerative colitis, its decrease in this study is consistent with previous reports on DSS-induced colitis (Wang et al., 2023). Importantly, CRF-H pretreatment attenuated these compositional shifts, partially reversing the DSS-induced alterations (Figure 5H).

At the genus level, the relative abundance of 15 top bacterial taxa was analyzed to evaluate the specific microecological regulatory effects of CRF-H (Figure 5G). DSS treatment severely impaired the intestinal symbiotic environment compared to that observed in the NC group, characterized by substantial depletion of dominant commensal *Lactobacillus*, and *unclassified Muribaculaceae* and *Lachnospiraceae_NK4A136_group*. Concurrently, the DSS model group exhibited increased relative abundances of opportunistic pathogens and inflammation-associated genera including *Bacteroides*, *Erysipelatoclostridium* (Figures 5I,J), and *Odoribacte*r. In agreement with previous findings, a decrease in relative abundance of *Lactobacillus* and an elevation in that of *Bacteroides* were observed in DSS-induced mice (Gao et al., 2025). Notably, a relatively high abundance of *Bacteroides* was observed in the sample NC5. However, this was accompanied by the highest recorded relative abundance of *Lachnospiraceae_NK4A136_group* across all samples, a taxon frequently linked to butyrate production and anti-inflammatory properties (Chen et al., 2021). Furthermore, typical pathogenic markers linked to severe DSS-induced mucosal damage, such as *Staphylococcus* and *Erysipelatoclostridium* (Zha et al., 2020), were virtually undetectable in this sample. These results show that the mouse gut microbiome is naturally different, and the NC group serves as a reliable, non-inflamed baseline for later tests. CRF-H did not directly preserve the relative abundance of *Lactobacillus*. Instead, it facilitated the recovery of other core commensals, which demonstrated relatively high relative abundances of *Bifidobacterium*, *Dubosiella*, and *Faecalibaculum* (Figures 5K–M). This compositional shift suggests that CRF-H may promote relative enrichment of specific beneficial commensals, potentially occupying ecological niches vacated by DSS-depleted taxa; however, further functional assays are necessary to verify these ecological dynamics.

### 3.8 CRF modulates specific microbial biomarkers strongly correlated with host colonic phenotypes

**Figure 6:**
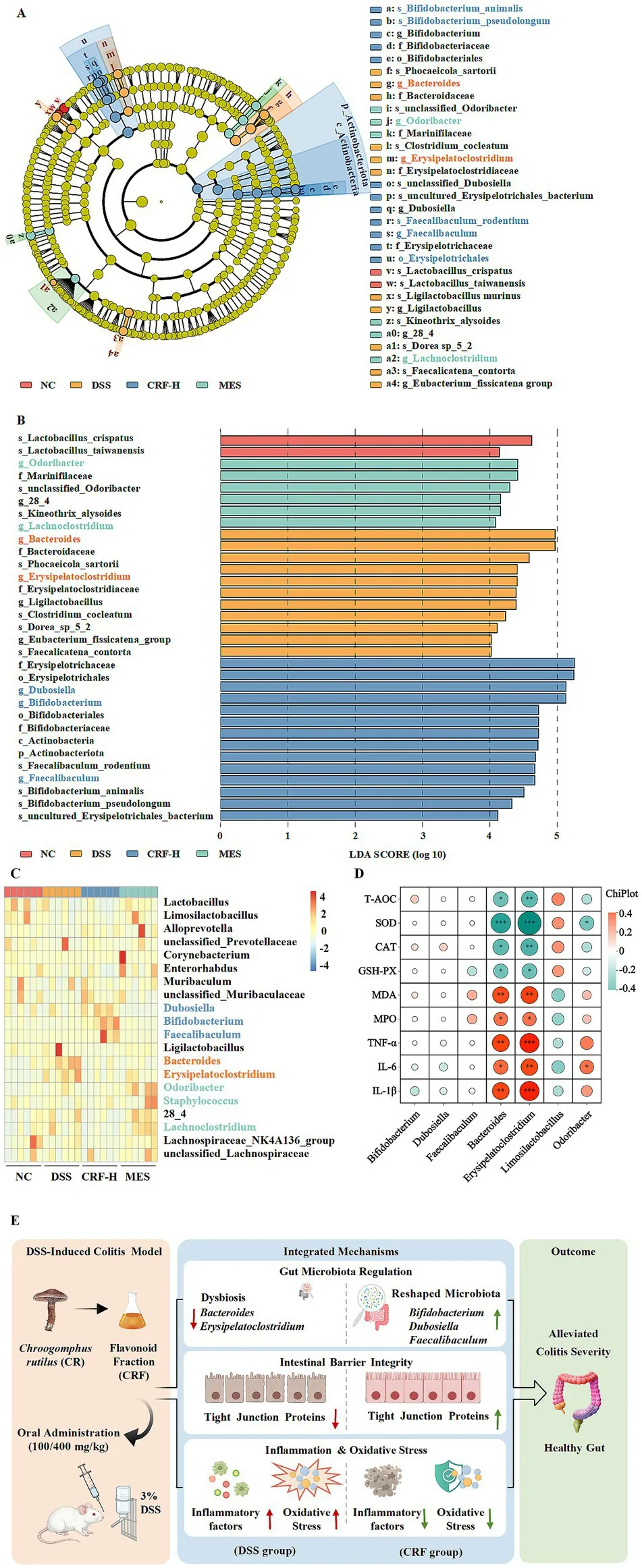
CRF-H pretreatment alters microbial biomarkers and host–microbiota crosstalk. **(A)** Cladogram of phylogenetic differences. **(B)** LEfSe bar chart (LDA > 4.0) of discriminant taxa. **(C)** Heatmap of differential genus. **(D)** Spearman correlations between microbial genus and host traits (^*^p < 0.05, ^**^p < 0.01). For **(A–D)**, *n* = 6 mice per group. (**E**) Schematic of the microbiota-inflammation-oxidative stress axis.

To identify differentially abundant taxa and explore the underlying regulatory network, linear discriminant analysis effect size (LEfSe) and hierarchical clustering analyses were performed. The gut microbiota of DSS-treated mice showed a pathological bloom of opportunistic pathogens and pro-inflammatory taxa (Figures 6A,B). Specifically, members of the family *Bacteroidaceae* (genus *Bacteroides*) and the family *Erysipelatoclostridiaceae* (genus *Erysipelatoclostridium*) were markedly enriched in the DSS group. These bacteria are linked to mucosal injury and are involved in mucus degradation and local inflammation (Zafar and Saier, 2021). MES and CRF-H pretreatment reduced the abundance of this harmful group of microbes, as shown by the heatmap rows that changed from red (high abundance) to blue (low abundance) in Figure 6C. The MES and CRF-H groups exhibited distinct microbial profiles. The relative abundance of *Odoribacter* and *Staphylococcus* was elevated in the MES group. MES exerts local anti-inflammatory effects, which may indirectly facilitate the recovery of certain commensals (e.g., *Lachnospiraceae*). However, in the absence of direct microbial competition, this immune-mediated recovery could render ecological niches susceptible to colonization by opportunistic pathogens such as *Staphylococcus* (Yan et al., 2025).

In contrast, CRF-H preconditioning suppressed the expansion of these detrimental taxa. Rather than simply restoring the microbiota to the NC baseline configuration, CRF-H remodeled it into a distinct, protective community. LEfSe analysis identified the key changes. CRF-H significantly enriched members of the phylum Actinobacteriota, ranging from the order Bifidobacteriales to the genus *Bifidobacterium*. These included the probiotic species *Bifidobacterium animalis* and *Bifidobacterium pseudolongum* (Figures 6A,B). Concurrently, a protective lineage within the order Erysipelotrichales was enriched, dominated by *Dubosiella* and *Faecalibaculum*, particularly *Faecalibaculum rodentium*. In the species abundance heatmap, these enriched microbes formed a distinct high-abundance cluster that was absent in the other groups (Figure 6C). These findings indicate that CRF modulates the gut microbiota in DSS-induced colitis. To assess the potential protective role of this microbial modulation, Spearman correlation analysis was performed between core taxa and host parameters (Figure 6D). Pathobionts such as *Bacteroides* and *Erysipelatoclostridium* were enriched in the DSS group, and they positively correlated with proinflammatory cytokines such as TNF-*α*, IL-6, and IL-1*β* (*p* < 0.05). These microbes also showed positive correlations with oxidative markers such as MPO and MDA. Conversely, the promoted beneficial commensals, including *Bifidobacterium*, *Dubosiella*, and *Faecalibaculum*, exhibited significant positive correlations with antioxidant enzymes (T-AOC, SOD, CAT, GSH-Px) and negative correlations with all measured inflammatory mediators. Although some individual correlations did not reach statistical significance, the overall pattern suggests that these beneficial taxa may not interact directly with host receptors but instead exert protective effects indirectly. *Bifidobacterium* and *Faecalibaculum rodentium* are known to ferment dietary fibers and produce short-chain fatty acids (SCFAs) such as acetate and butyrate (Chen Y. et al., 2025). SCFAs are essential for fueling colonocytes, reinforcing tight junctions, and promoting regulatory T-cell differentiation, thereby suppressing intestinal inflammation (Nogal et al., 2021). We hypothesize that these beneficial taxa promote SCFA production through cross-feeding interactions, although SCFA levels were not measured in this study, representing a limitation. Overall, these correlation analyses support the hypothesis that CRF-H exerts protective effects against colitis partly by promoting beneficial microbes and suppressing pathobionts, thereby attenuating inflammation and enhancing antioxidant capacity (Figure 6E). However, these findings are based on 16S rRNA gene sequencing, which yields relative abundance data rather than direct functional evidence. Accordingly, the proposed microbiota-mediated mechanisms remain correlative and require further validation through metagenomic, metabolomic, or interventional approaches.

## 4 Conclusion

Our findings indicate that CRF pretreatment attenuates DSS-induced colitis in mice, as reflected by reduced colonic oxidative stress, preserved tight junction protein expression, suppressed systemic inflammation, and modulation of gut microbiota composition. These results support the hypothesis that CRF may exert protective effects through a multi-target mechanism involving anti-inflammatory, antioxidant, and microbiota-modulating activities. However, the bioavailability of individual flavonoids in CRF has not been characterized. These findings remain mechanistic hypotheses rather than established causal pathways. Future studies using purified compounds, pathway-specific inhibitors, and pharmacokinetic analyses are needed to establish causative mechanisms and identify the active principle(s) responsible for the observed benefits.

## Data Availability

The data presented in this study can be found in the OMIX database, hosted by the National Genomics Data Center (NGDC) of China, under accession number OMIX018713.
