# Supplementary material for: Flavonoids from Chroogomphus rutilus attenuate DSS-induced colitis, exhibiting anti-inflammatory, antioxidant, and gut microbiota-modulating effects
Source: Front Microbiol. 2026 Aug 10;17:1886708. doi: 10.3389/fmicb.2026.1886708 (PMC13500521; doi:10.3389/fmicb.2026.1886708)
Supplement: Supplementary file 1 [file Table_1.docx]

Supplementary Material

# 1 Supplementary Data

A comprehensive profiling of flavonoid compounds in CRF was performed using LC-MS/MS. The representative total ion chromatogram (TIC) is shown in Figure S1. Most of the tentatively identified compounds were flavonoids, as summarized in Table S1. These included 5-demethylnobiletin, chrysin, catechin, phloridzin, daidzin, oroxindin, phloretin, daidzein, skullcapflavone II, 10-gingerol, baicalin, diosmetin 7-O-rutinoside, cynaroside, 7-methoxyflavonol, isoquercetin, nardosinone, and 5,7-dihydroxy-3-phenyl-4H-chromen-4-one. Additionally, several other flavonoid glycosides such as quercetin-3-O-galactoside, apigenin-7-O-glucuronide, and diosmetin 7-O-rutinoside were also detected.

# 2 Supplementary Figures and Tables

## 2.1 Supplementary Figures

## （A）

（B）

## **Supplementary Figure S1.** Combined LC-MS/MS base peak chromatograms (BPC) of CRF detected by UPLC-Q-TOF-MS/MS in both positive and negative ion modes. The complementary ionization coverage provided by the negative ion mode (A) and positive ion mode (B) was used to maximize the detection of structurally diverse flavonoids.

## 2.2 Supplementary Tables

Mass spectrometry parameters of CRF.

Note: Compounds were identified using an AB SCIEX TripleTOF 5600 system operated in information-dependent acquisition (IDA) mode. In each acquisition cycle, the most intense precursor ions (>100 cps) were automatically selected for MS/MS fragmentation at a collision energy of 30 eV, with up to 15 MS/MS spectra collected per cycle. Raw data were processed using MSDIAL version 4.6 for peak detection, alignment, and spectral matching against an integrated database comprising Metlin, MassBank, MoNA, and HMDB (version V6.0). Identification was based on both MS1 accurate mass (tolerance < 5%) , with an identification score cut-off of 60%. Precursor ion m/z, adduct type, and molecular formula are provided for each identified compound.

Table S1. Mass spectrometry parameters of CRF

| NO | Peak time  (min) | Adduct | Precursor (m/z) | Major product ions  (m/z) | Molecmlar formmla | Compounds | Reference |
| --- | --- | --- | --- | --- | --- | --- | --- |
| 1 | 0.661867 | [M-H]- | 387.10 | 374,359 | C20H20O8 | 5-Demethylnobiletin | Wang et al., 2021 |
| 2 | 0.837667 | [M-H]- | 253.05 | 225,209,181 | C15H10O4 | Chrysin | Wen et al., 2023 |
| 3 | 6.638633 | [M-H]- | 289.07 | 245,205,179 | C15H14O6 | Catechin | Dong et al., 2024 |
| 4 | 7.473617 | [M-H]- | 463.09 | 301,179,151 | C21H20O12 | Quercetin-3-O-galactoside | Zhao et al., 2018 |
| 5 | 9.09965 | [M-H]- | 435.13 | 273, 167，151 | C21H24O10 | Phloridzin | Kolniak et al., 2013 |
| 6 | 10.54987 | [M-H]- | 415.10 | 253,191,155 | C21H20O9 | Daidzin | Li et al., 2024 |
| 7 | 10.63777 | [M-H]- | 445.08 | 269,241,175 | C21H18O11 | apigenin-7-O-glucuronide | Wen et al., 2023 |
| 8 | 12.17588 | [M-H]- | 459.09 | 283,268,255 | C22H20O11 | Oroxindin | Liu et al., 2020 |
| 9 | 12.26378 | [M-H]- | 273.08 | 167,149,125 | C15H14O5 | Phloretin | Zhu et al., 2022 |
| 10 | 15.95527 | [M-H]- | 253.05 | 227,199,137 | C15H10O4 | Daidzein | Nakata et al., 2018 |
| 11 | 16.26288 | [M-H]- | 373.099 | 343,328,285 | C19H18O8 | skullcapflavone II | Wen et al., 2023 |
| 12 | 17.80103 | [M-H]- | 349.24 | 193, 179, 155 | C21H34O4 | 10-Gingerol | Tao et al., 2009 |
| 13 | 20.43783 | [M-H]- | 445.10 | 269,241,175 | C21H18O11 | Baicalin | Wen et al., 2023 |
| 14 | 21.09705 | [M-H]- | 607.40 | 299,284,151 | C28H32O15 | Diosmetin 7-O-rutinoside | Abdelaziz et al., 2020 |
| 15 | 21.31678 | [M-H]- | 447.11 | 285,151,133 | C21H20O11 | Cynaroside | Lin & Harnly, 2007 |
| 16 | 1.046567 | [M+H]^+^ | 269.1081 | 251, 241, 223 | C16H12O4 | 7-Methoxyflavonol |  |
| 17 | 7.329117 | [M+H]^+^ | 465.11 | 301,179,151 | C21H20O12 | Isoquercetin | Lin & Harnly, 2007 |
| 18 | 12.25622 | [M+H]^+^ | 251.16 | 233,205,191 | C15H22O3 | Nardosinone | Xue et al., 2023 |
| 19 | 15.59975 | [M+H]+ | 255.07 | 225,191,155 | C15H10O4 | 5,7-dihydroxy-3-phenyl-4H-chromen-4-one | Li et al., 2024 |

Footnote: Precursor ion mass accuracy was within 5 ppm for all compounds. Elemental formulas were verified by MS1 isotopic pattern matching (tolerance < 5%). Major product ions were selected from raw MS/MS spectra based on highest intensities. References support fragment assignments.

**Reference：**

Abdelaziz, S., Al Yousef, H. M., Al-Qahtani, A. S., Hassan, W. H. B., Fantoukh, O. I., & El-Sayed, M. A. (2020). Phytochemical profile, antioxidant and cytotoxic potential of Parkinsonia aculeata L. growing in Saudi Arabia. *Saudi Pharmaceutical Journal*, *28*(9), 1129-1137. <https://doi.org/10.1016/j.jsps.2020.08.001>

Dong, S., Ding, L., Zheng, X., Wang, O., & Cai, S. (2024). Phenolic Compositions of Different Fractions from Coffee Silver Skin and Their Antioxidant Activities and Inhibition towards Carbohydrate-Digesting Enzymes. *Foods*, *13*(19), 3083. <https://doi.org/10.3390/foods13193083>

Kolniak-Ostek, J., Oszmianski, J., & Wojdylo, A. (2013). Effect of l-ascorbic acid addition on quality, polyphenolic compounds and antioxidant capacity of cloudy apple juices. *European Food Research and Technology*, *236*(5), 777-798. <https://doi.org/10.1007/s00217-013-1931-z>

Li, H. R., Wang, H., Dong, P. P., Li, H. J., Wang, S. P., & Zhang, J. Y. (2024). Comparative characterization of the metabolites of phloretin and phlorizin in rats using UHPLC-Q-Exactive Orbitrap mass spectrometer. *Arabian Journal of Chemistry*, *17*(3), 13, Article 105597. <https://doi.org/10.1016/j.arabjc.2023.105597>

Lin, L. Z., & Harnly, J. M. (2007). A screening method for the identification of glycosylated flavonoids and other phenolic compounds using a standard analytical approach for all plant materials. *Journal of Agricultural and Food Chemistry*, *55*(4), 1084-1096. <https://doi.org/10.1021/jf062431s>

Liu, Q., Zuo, R., Wang, K., Nong, F. F., Fu, Y. J., Huang, S. W., et al. (2020). Oroxindin inhibits macrophage NLRP3 inflammasome activation in DSS-induced ulcerative colitis in mice via suppressing TXNIP-dependent NF-κB pathway. *Acta Pharmacologica Sinica*, *41*(6), 771-781. <https://doi.org/10.1038/s41401-019-0335-4>

Nakata, R., Yoshinaga, N., Teraishi, M., Okumoto, Y., Huffaker, A., Schmelz, E. A., et al. (2018). A fragmentation study of isoflavones by IT-TOF-MS using biosynthesized isotopes. *Bioscience Biotechnology and Biochemistry*, *82*(8), 1309-1315. <https://doi.org/10.1080/09168451.2018.1465810>

Tao, Y., Li, W. K., Liang, W. Z., & Van Breemen, R. B. (2009). Identification and Quantification of Gingerols and Related Compounds in Ginger Dietary Supplements Using High-Performance Liquid Chromatography-Tandem Mass Spectrometry. *Journal of Agricultural and Food Chemistry*, *57*(21), 10014-10021. <https://doi.org/10.1021/jf9020224>

Wang, K., Tian, J., Li, Y., Liu, M., Chao, Y., Cai, Y., et al. (2021). Identification of Components in Citri Sarcodactylis Fructus from Different Origins via UPLC-Q-Exactive Orbitrap/MS. *ACS Omega*, *6*(26), 17045-17057. <https://doi.org/10.1021/acsomega.1c02124>

Wen, X. Q., Xie, W. W., Gao, J., Zhang, D. D., Yang, M. X., Zhang, Z. Q., et al. (2023). Systematically uncovering the absorbed effective substances of Radix Scutellaria-licorice drug pair in rat plasma against COVID-19 using a combined UHPLC-Q-TOF-MS analysis and target network pharmacology. *PloS One*, *18*(8), 21, Article e0289121. <https://doi.org/10.1371/journal.pone.0289121>

Xue, B. X., Yang, T. T., He, R. S., Gao, W. K., Lai, J. X., Liu, S. X., et al. (2023). Degradation Profiling of Nardosinone at High Temperature and in Simulated Gastric and Intestinal Fluids. *Molecules*, *28*(14), 23, Article 5382. <https://doi.org/10.3390/molecules28145382>

Zhao, W., Shang, Z., Li, Q., Huang, M., He, W., Wang, Z., et al. (2018). Rapid Screening and Identification of Daidzein Metabolites in Rats Based on UHPLC-LTQ-Orbitrap Mass Spectrometry Coupled with Data-Mining Technologies. *Molecules*, *23*(1), 151. <https://doi.org/10.3390/molecules23010151>

Zhu, Y. D., Wang, W. X., Huang, Q. J., Hu, C. L., & Sang, S. M. (2022). Metabolic Investigation on the Interaction Mechanism between Dietary Dihydrochalcone Intake and Lipid Peroxidation Product Acrolein Reduction. *Molecular Nutrition & Food Research*, *66*(9), 14, Article 2101107. <https://doi.org/10.1002/mnfr.202101107>

Table S2. Intersection targets of CRF and UC.

| NO | Gene symbol |
| --- | --- |
| 1 | AKR1B1 |
| 2 | TNF |
| 3 | IL2 |
| 4 | XDH |
| 5 | ADRA2A |
| 6 | ACHE |
| 7 | RPS6KA3 |
| 8 | CA2 |
| 9 | CA4 |
| 10 | ALDH2 |
| 11 | EGFR |
| 12 | CA1 |
| 13 | CA9 |
| 14 | PTGS2 |
| 15 | CD38 |
| 16 | TP53 |
| 17 | SLC29A1 |
| 18 | AKR1B10 |
| 19 | MMP13 |
| 20 | MMP2 |
| 21 | MMP12 |
| 22 | APP |
| 23 | ELANE |
| 24 | SLC37A4 |
| 25 | PRKCD |
| 26 | PRKCA |
| 27 | EDNRA |
| 28 | ABCB1 |
| 29 | NEU4 |
| 30 | ALOX5 |
| 31 | TERT |
| 32 | ADORA3 |
| 33 | PTPN22 |
| 34 | PGF |
| 35 | VEGFA |
| 36 | MMP9 |
| 37 | PTGS1 |
| 38 | BCL2 |
| 39 | KIT |
| 40 | KDR |
| 41 | MAPT |
| 42 | PGD |
| 43 | FUT7 |
| 44 | FUT4 |
| 45 | FGFR1 |
| 46 | SRC |
| 47 | MET |
| 48 | POLB |
| 49 | PLA2G2A |
| 50 | PLA2G10 |
| 51 | CYP1B1 |
| 52 | HIF1A |
| 53 | MMP14 |
| 54 | ERBB2 |
| 55 | MAPK14 |
| 56 | FLT1 |
| 57 | DNMT1 |
| 58 | ESR1 |
| 59 | ESR2 |
| 60 | DYRK1A |
| 61 | STAT1 |
| 62 | LDHA |
| 63 | COMT |
| 64 | BCL2L1 |
| 65 | IGF1R |
| 66 | ALK |
| 67 | SERPINE1 |
| 68 | AKR1C3 |
| 69 | GPR35 |
| 70 | ALB |
